# Rare bacterial biosphere is more environmental controlled and deterministically governed than abundant one in sediment of thermokarst lakes across the Qinghai-Tibet Plateau

**DOI:** 10.3389/fmicb.2022.944646

**Published:** 2022-07-25

**Authors:** Ze Ren, Wei Luo, Cheng Zhang

**Affiliations:** ^1^Research and Development Center for Watershed Environmental Eco-Engineering, Advanced Institute of Natural Sciences, Beijing Normal University, Zhuhai, China; ^2^School of Environment, Beijing Normal University, Beijing, China; ^3^Key Laboratory for Polar Science, Polar Research Institute of China, Ministry of Natural Resources, Shanghai, China; ^4^School of Oceanography, Shanghai Jiao Tong University, Shanghai, China; ^5^School of Engineering Technology, Beijing Normal University, Zhuhai, China

**Keywords:** thermokarst lakes, sediment, abundant and rare taxa, assembly mechanisms, Qinghai-Tibet Plateau

## Abstract

Thermokarst lakes are widely distributed in cold regions as a result of ice-rich permafrost thaw. Disentangling the biogeography of abundant and rare microbes is essential to understanding the environmental influences, assembly mechanisms, and responses to climate change of bacterial communities in thermokarst lakes. In light of this, we assessed the abundant and rare bacterial subcommunities in sediments from thermokarst lakes across the Qinghai-Tibet Plateau (QTP). The operational taxonomic unit (OTU) richness was more strongly associated with location and climate factors for abundant subcommunities, while more strongly associated with physicochemical variables for rare subcommunities. The relative abundance of abundant and rare taxa showed opposite patterns with abundant taxa having greater relative abundance at higher latitude and pH, but at lower mean annual precipitation and nutrients. Both the abundant and rare subcommunities had a clear distribution pattern along the gradient of latitude and mean annual precipitation. Abundant subcommunities were dominantly shaped by dispersal limitation processes (80.9%), while rare subcommunities were shaped almost equally by deterministic (47.3%) and stochastic (52.7%) processes. The balance between stochastic and deterministic processes was strongly environmentally adjusted for rare subcommunities, while not associated with environmental changes for abundant subcommunities. The results shed light on biogeography patterns and structuring mechanisms of bacterial communities in thermokarst lakes, improving our ability to predict the influences of future climate change on these lakes.

## Introduction

Thermokarst lakes and ponds are the most common type of aquatic ecosystems in the Arctic regions (Farquharson et al., 2016; In'T Zandt et al., 2020) and are also extensively distributed in the Qinghai-Tibet Plateau (QTP) (Luo et al., 2015, 2020; Zhang et al., 2017). These lakes are formed as a consequence of ice-rich permafrost thaw, producing widespread sediments and sedimentary structures underneath the thermokarst lakes (Murton and Fard, 2001; Wetterich et al., 2008; de Jong et al., 2018). Surrounding permafrost is continuously delivered to the lake due to bank abrasion and collapse, supplementing lake sediments (Kokelj and Jorgenson, 2013; Turetsky et al., 2019). Global warming has accelerated permafrost degradation, converting vast permafrost to the sediment of thermokarst lakes (Edwards et al., 2016; Biskaborn et al., 2019). These anaerobic and organic matter-rich sediment is an active area of carbon cycling, and biogeochemical hotspots of other elements (Du Toit, 2018; In'T Zandt et al., 2020; Jongejans et al., 2021). Sediments host a huge number of versatile microorganisms driving vital biogeochemical processes (Steger et al., 2011; Ren et al., 2022a), such as the metabolism of carbon dioxide (CO_2_) and methane (CH_4_) (Matheus Carnevali et al., 2018; Jongejans et al., 2021). Therefore, a shift in bacterial communities may be one of the most sensitive indicators of environmental changes in the lake and results in significant impacts on regional and global biogeochemical processes. However, the community assembly of microbial communities in the sediment of thermokarst lakes is not clear, particularly across a large spatial scale, such as the QTP in this study.

In lake ecosystems, bacterial communities exhibit high diversities and variabilities, with a small fraction of high-abundant dominant taxa coexisting with a considerable proportion of low-abundant rare taxa (Lynch and Neufeld, 2015; Jia et al., 2018). Although present in lower abundances, rare taxa can have a disproportionately large functional effect in ecosystems. Previous studies have indicated that abundant and rare taxa exhibit fundamentally different characteristics and play different ecological roles (Logares et al., 2014; Lynch and Neufeld, 2015). Distinguishing biogeography, community assembly, and environmental response between abundant and rare microbes is crucial for understanding microbe-mediated ecological processes and functions. Therefore, increasing studies focused on the biogeography of abundant and rare biosphere in various environments, such as agricultural soil (Jiao and Lu, 2019; Xue et al., 2020; Gschwend et al., 2021), freshwater (Zhang et al., 2018a; Ren et al., 2020; Nyirabuhoro et al., 2021), and ocean (Campbell et al., 2011; Logares et al., 2014; Ruiz González et al., 2019).

Microorganisms are the most diverse group of life on the planet, and it is a long-standing puzzle for microbiologists that how such high diversity is generated and maintained (Stegen et al., 2012; Zhou and Ning, 2017). Various deterministic factors and stochastic processes are thought important in controlling microbial diversity and community assembly (Zhou et al., 2014; Stegen et al., 2015; Aguilar and Sommaruga, 2020). Moreover, it has also been widely studied recently that community assembly processes, including deterministic and stochastic processes, can impose substantial influences on biogeochemical functions, due to the fact that species richness and abundance are determinants of ecosystem functions (Graham et al., 2016; Graham and Stegen, 2017; Le Moigne et al., 2020; Luan et al., 2020). For example, stochastic processes may suppress organic carbon metabolism (Le Moigne et al., 2020; Luan et al., 2020). In our previous studies of thermokarst lakes, we found that abundant and rare bacterial taxa structure differently in sediment and water in thermokarst lakes in the Yellow River source area (Ren et al., 2022a). Across a large spatial scale, more environmental variables would be important in shaping bacterial communities, such as spatial and climatic factors (Picazo et al., 2020; Zhang et al., 2020). However, little is known about ecological assembly mechanisms in shaping the biogeography of abundant and rare taxa in thermokarst lakes across a large spatial scale thermokarst landscape.

On the QTP, over 40% of the land area is covered by permafrost (Zou et al., 2017), which is sensitive to climate warming. The QTP is warming faster than other areas of the earth (Yao et al., 2000), resulting in extensive changes in thermokarst lakes with increasing lake numbers and expansion of lake areas (Luo et al., 2015; Zhang et al., 2017). In this study, we investigated bacterial communities in sediments from 44 thermokarst lakes across the QTP. The objectives of this study were to assess the distribution patterns, assembly mechanisms, and influencing environmental variables of abundant and rare subcommunities in thermokarst lake sediments across the QTP. The results can enrich our understanding of biogeography patterns of bacterial communities in these lakes and improve our ability to predict the influences of future climate change.

## Methods

### Study area, field sampling, and chemical analysis

**Figure 1:**
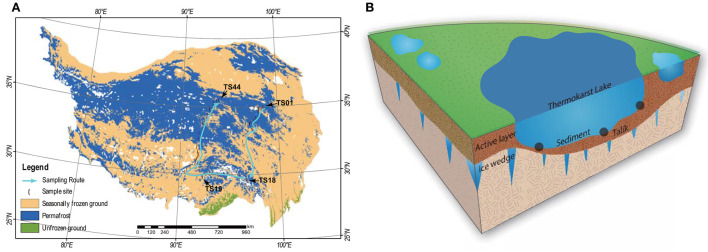
**(A)** Maps of the 44 sampling sites of thermokarst lake sediment across the Qinghai-Tibet Plateau. The distribution of the permafrost was cited by Zou et al. (2017). The map was modified by Ren et al. (2022c). **(B)** A schematic view of the thermokarst lake and the sampling strategy.

In this study, a total of 44 thermokarst lakes (TS01–TS44) were sampled in July 2021 on the QTP (Figure 1). The investigated lakes are distributed in an extensive area from the longitude (LON) of 90.6E to 98.6E and from the latitude (LAT) of 30.2N to 35.0N. The elevation (ELE) of the samples ranged from 3,569 to 4,959 m above sea level. The studied lakes are all located within permafrost landscapes and are closed water bodies without inflow and outflow. The QTP has the largest areas of permafrost in the mid- and low-elevation regions of the world, with an area of 1.06 × 10^6^ km^2^ (Zou et al., 2017). The mean annual temperature (MAT) and mean annual precipitation (MAP) of our study regions were extracted from the spatial dataset of the QTP climate ranging from 1991 to 2020 (Zhou, 2018), which was downloaded from the National Tibetan Plateau Data Center (https://data.tpdc.ac.cn/en/). In each lake, the top 15 cm of the sediment was collected from 3 points. The sediment bacterial sample was collected in a 45-ml sterile centrifuge tube and reserved in liquid nitrogen immediately. The remaining sediment was freeze-dried for physicochemical analyses. pH was measured in a 1:2.5 dry sediment to distilled water ratio using a pH meter and conductivity was measured in a 1:5 dry sediment to distilled water ratio using a conductivity meter. Sediment organic carbon (SOC) was measured by the potassium dichromate oxidation spectrophotometric method (Chinese standard method HJ615-2011) (Wang et al., 2020; Ren et al., 2022b). Total nitrogen (TN) was measured using the modified Kjeldahl method (Chinese standard method HJ717-2014) (Wang et al., 2020; Ren et al., 2022b). Total phosphorus (TP) was measured using the ascorbic acid colorimetric method after microwave extraction with nitric acid (Dancer et al., 1998). The basic information about the lakes is given in Supplementary Table 1.

### DNA extraction, PCR, and sequencing

Deoxyribonucleic acid (DNA) was extracted from sediment using the HiPure Soil DNA Kit (Magen, China) following the manufacturer's protocols. The V3–V4 hypervariable regions of prokaryotic 16S ribosomal RNA (rRNA) were amplified using the universal forward primer (5'-TACGGRAGGCAGCAG-3') and reverse primer (5'-AGGGTATCTAATCCT-3') (Nossa et al., 2010). For each sample, three replicates of PCR amplifications were performed to minimize amplification bias. Then, the triplicate PCR products were combined, purified, and quantified. The DNA libraries were multiplexed and loaded on an Illumina MiSeq platform (Illumina, San Diego, CA, USA) according to the manufacturer's instructions. Raw sequences were analyzed using QIIME version 1.9.1 (Caporaso et al., 2010). The effective sequences were clustered into operational taxonomic units (OTUs) at 97% sequence identity against the SILVA 138 database (Quast et al., 2013). The singleton OTUs were removed and the sequences were normalized to the same depth of 24,251 sequences per sample to avoid the bias of surveying efforts. 16S rRNA gene sequences were uploaded to the China National Center for Bioinformation (PRJCA009850, CRA007082).

### Analyses

Microbial taxa were commonly defined as abundant and rare according to their relative abundance (Pedros-Alio, 2012; Logares et al., 2014). In this study, abundant and rare taxa were identified at the local level (in one sample) as the OTUs with a relative abundance of ≥ 0.1 and < 0.01% in each sample, respectively (Jiao and Lu, 2020; Ren and Gao, 2022). The abundant and rare subcommunities were identified at the regional level (in the metacommunities including all the samples) (Jiao and Lu, 2020; Ren and Gao, 2022). The abundant subcommunities were composed of the OTUs with an average relative abundance of ≥0.1% across all the samples and the rare subcommunities were composed of the OTUs with an average relative abundance of <0.01% across all the samples. The difference in OTU richness between abundant and rare taxa was assessed using the Wilcoxon rank-sum test. The relationships between OTU richness and relative abundance vs. environmental variables were assessed using Spearman's correlation. The β-diversity was calculated as the Bray–Curtis distance based on the relative abundance of OTUs. The difference in the β-diversity between abundant and rare subcommunities was assessed using the Wilcoxon rank-sum test. Non-metric multidimensional scaling (NMDS) analysis was performed to assess the distribution of abundant and rare subcommunities along latitude and MAP gradients using the vegan 2.5-7 package (Oksanen et al., 2020). Structural equation modeling (SEM) analysis was conducted to depict the relationships between location, climate, and physicochemical environments, and also abundant and rare subcommunities. In SEM, environmental factors were reduced in dimensions using principal component analysis (PCA), and the first axis of PCA was used. The location factor includes latitude, longitude, and elevation. The climate factor includes mean annual temperature and mean annual precipitation. The nutrient factor includes sediment organic carbon, total nitrogen, and total phosphorus. The nutrient ratios factor includes C:N, C:P, and N:P ratios. Abundant and rare subcommunities were reduced in dimension using NMDS and the first axis of NMDS was used. SEMs were conducted using the R package lavaan 0.6–10 (Rosseel, 2012).

The assembly processes of abundant and rare subcommunities were estimated using a null model analysis in the Picante version 1.8.2 package in R (Kembel et al., 2010). The phylogenetic tree was built by the neighbor-joining method. The beta mean nearest taxon distance (βMNTD) was calculated to quantify the turnover in phylogenetic composition between any paired communities. Then, the beta nearest taxon index (βNTI) was calculated by evaluating the difference between the observed βMNTD and the mean of the null distribution of βMNTD in units of SD. Different deterministic processes were estimated with βNTI values < −2 or > +2 indicating heterogeneous selection and homogenous selection, respectively (Stegen et al., 2013; Zhou and Ning, 2017). Stochastic processes were falling within −2 < βNTI < 2. The relative influences of the stochastic processes were further estimated by incorporating the Bray–Curtis-based Raup–Crick metric (RC_Bray_). The pairwise comparisons with −2 < βNTI < 2 and RC_Bray_ < −0.95 were identified as homogeneous dispersal. Those with −2 < βNTI < 2 and RC_Bray_ > 0.95 were identified as dispersal limitation. Those with −2 < βNTI < 2 and −0.95 < RC_Bray_ < 0.95 were assigned to “undominated.” Both the βNTI and RC_Bray_ values were calculated with 999 permutations. The Mantel test was further used to assess the relationships between β-diversity and βNTI vs. environmental variables with 999 permutations. All the statistical analyses were carried out in R 4.1.2 (R Core Team, 2020).

## Results

### General distribution of abundant and rare taxa

**Figure 2:**
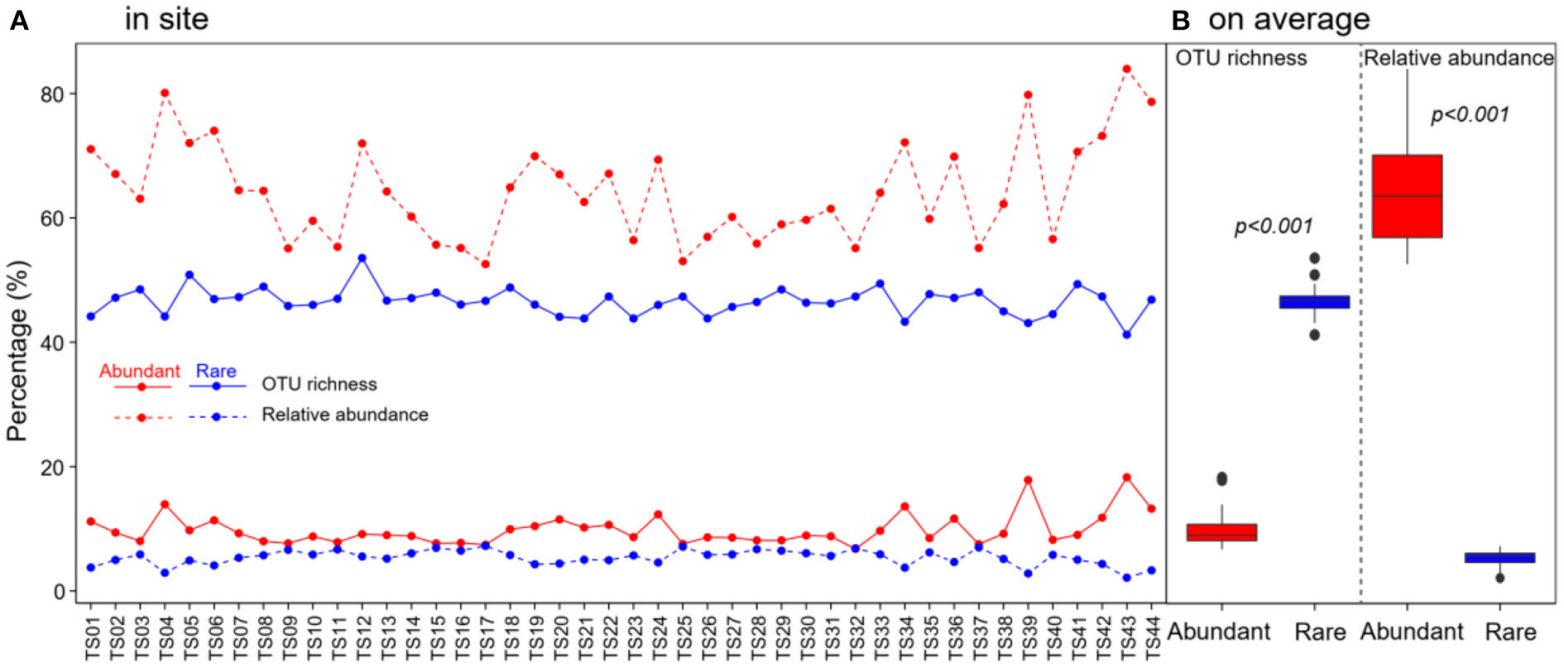
Distribution of OTU richness and relative abundance of abundant and rare bacterial taxa in thermokarst lake sediment **(A)** in each lake and **(B)** on average across the lakes.

**Figure 3:**
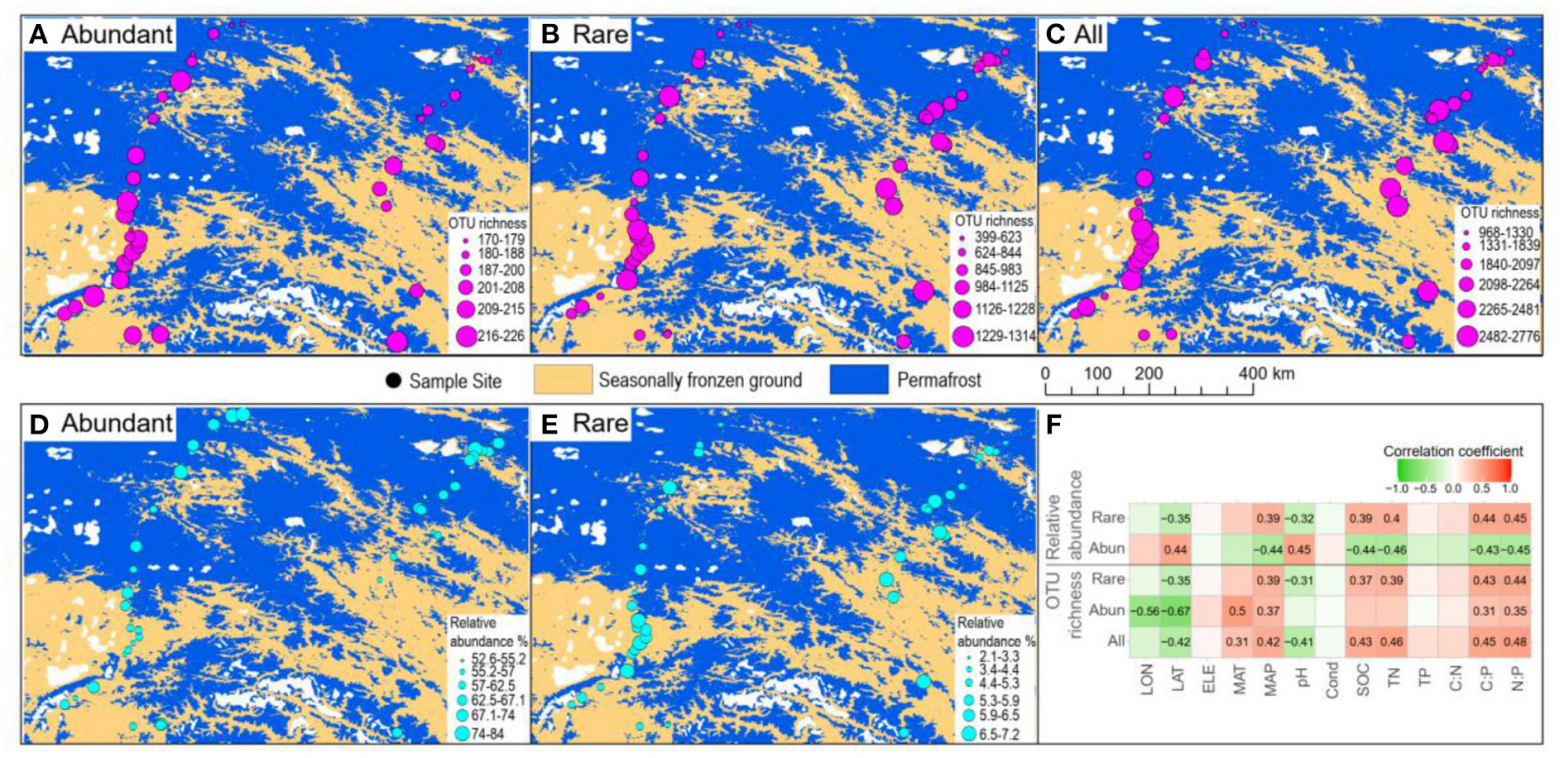
Spatial patterns of abundant and rare taxa. **(A–C)** Spatial distribution of the OTU richness in terms of abundant, rare, and all the taxa. **(D,E)** Spatial distribution of the relative abundance of abundant and rare taxa. **(F)** Spearman's correlation between OTU richness and relative abundance of abundant and rare taxa vs. environmental variables. Only significant (*p* < *0.05*) correlations are shown in the number, which is the correlation coefficient.

In total, 1,067,004 high-quality sequences were obtained after quality filtering and chimeric sequence removal. These sequences were clustered into 8,153 OTUs. As expected, abundant taxa only accounted for a very low proportion of the OTUs (9.8% on average), but a very high proportion of the sequences (64.3% of the average relative abundance) (Figure 2). On the contrary, rare taxa accounted for a high proportion of the OTUs (46.6% on average), but a very low proportion of the sequences (5.4% of the average relative abundance) (Figure 2). OTU richness of abundant taxa had strong correlations with spatial and climatic variables, with negative correlations with LON and LAT, while positive correlations with MAT and MAP, and positive correlations with C:P and N:P (Figure 3). However, OTU richness of rare taxa had strong correlations with physicochemical variables, including a negative correlation with pH, but positive correlations with SOC, TN, C:P, and N:P (Figure 3). In addition, OTU richness of rare taxa also negatively correlated with LAT and positively correlated with MAP (Figure 3). In terms of relative abundance, Spearman's correlation showed that abundant taxa had a greater relative abundance at higher latitude and pH and lower MAP, SOC, TN, C:P, and N:P (Figure 3), suggesting that abundant taxa had high adaptation to unfavorable environments. However, the relative abundance of rare taxa had completely opposite patterns compared to abundant taxa (Figure 3). The results indicate that the abundant and rare taxa had distinct distribution patterns and distinct environmental preferences in thermokarst lake sediment across the QTP.

### Community composition and beta diversity

**Figure 4:**
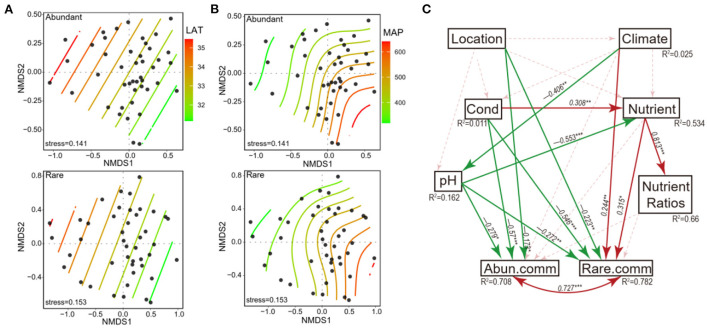
Non-metric multidimensional scaling (NMDS) ordination showing the distribution of abundant and rare subcommunities along the **(A)** latitude gradient and **(B)** mean annual precipitation gradient. The contour lines show the gradients. **(C)** Structural equation modeling analysis illustrating the relationships between location (including latitude, longitude, and elevation), climate (including mean annual temperature and mean annual precipitation), pH, conductivity, nutrient (including sediment organic carbon, total nitrogen, and total phosphorus), nutrient ratios (including C:N, C:P, and N:P ratios), and abundant and rare subcommunities. Solid and dashed arrows represent the significant and non-significant relationships, respectively. Red and green arrows represent positive and negative relationships, respectively. The significant path coefficient is shown adjacent to the path with *, **, and *** denote the significant level of *p* < 0.05, *p* < 0.01, and *p* < 0.001, respectively.

Abundant and rare subcommunities were mainly composed of Proteobacteria, Bacteroidota, and Acidobacteriota in terms of both the OTU richness and relative abundance of sequences. NMDS analysis showed that both the abundant and rare subcommunities had a clear distribution pattern along the gradient of LAT and MAP (Figure 4). The SEM results showed that both the abundant and rare subcommunities were significantly directly affected by location, pH, and conductivity (Figure 4). In addition, rare subcommunities were directly affected by climate and nutrients as well (Figure 4).

**Figure 5:**
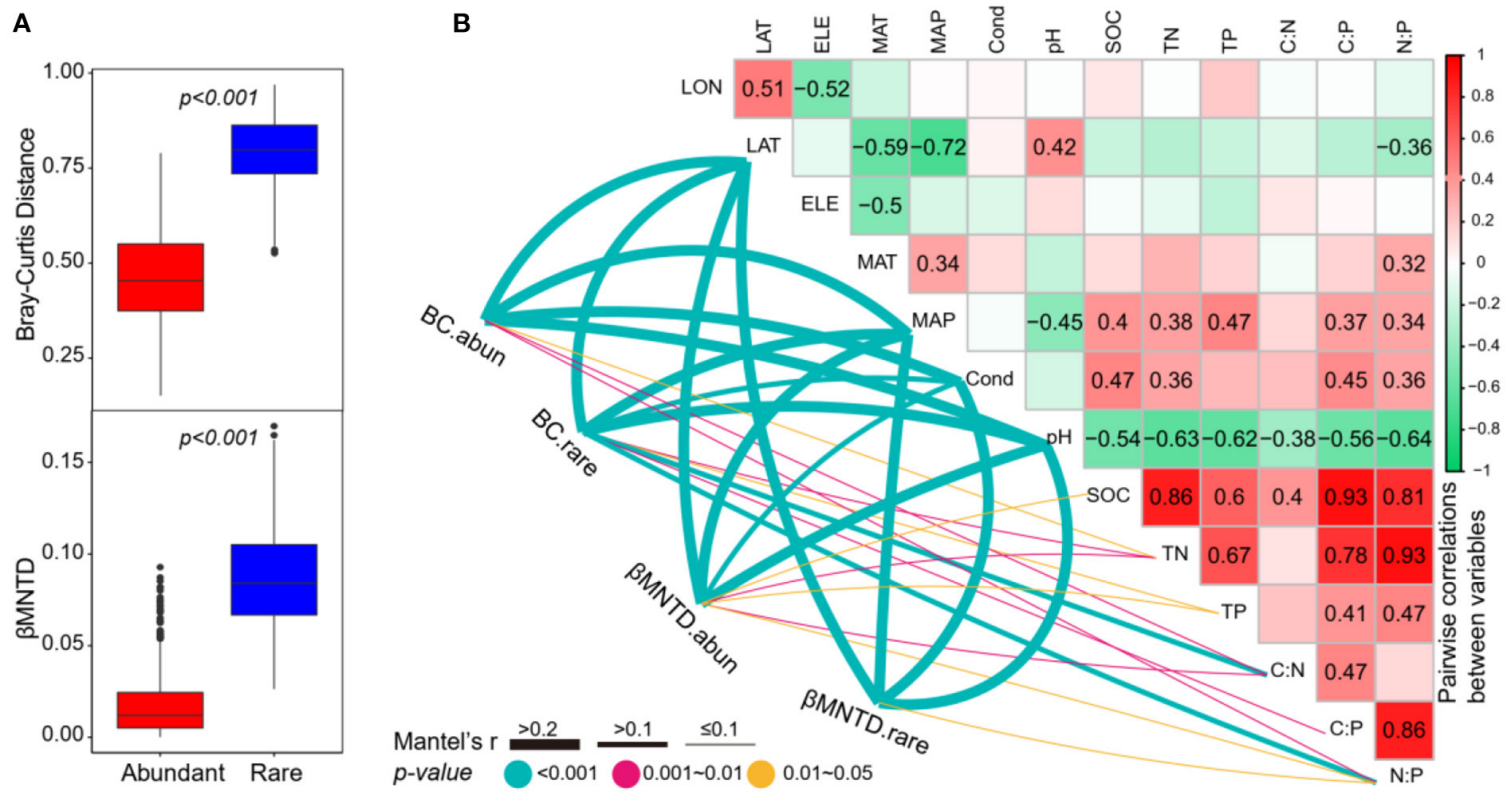
**(A)** The differences of the Bray–Curtis distance and βMNTD between abundant and rare subcommunities (Wilcoxon rank-sum test, *p* < 0.001). **(B)** Pairwise correlations between environmental variables, and the Mantel tests between environmental variables vs. β-diversity. BC.abun, BC.rare, βMNTD.abun, and βMNTD.rare represent the Bray–Curtis distance and βMNTD of abundant and rare subcommunities, respectively. The lines denote the significant relationships with line width representing the Mantel r statistic. Pairwise correlations between environmental variables are shown in the heatmap matrix denoting Pearson's correlation coefficient. The significant results are shown in numbers. The abbreviations of the environmental variables are explained in the Methods section.

As expected, the abundant subcommunities had a significantly lower taxonomic β-diversity (the Bray–Curtis distance) and phylogenetic β-diversity (βMNTD) than rare subcommunities (Figure 5A), suggesting that the rare subcommunities had a higher variation across the study lakes. For taxonomic β-diversity, the Mantel tests showed that both the abundant and rare subcommunities were significantly correlated to the divergences of latitude, MAP, conductivity, pH, TN, C:N, and N:P (Figure 5). Moreover, the taxonomic β-diversity of rare subcommunities was also significantly correlated to TP and C:P (Figure 5). For phylogenetic β-diversity, the Mantel tests showed that both the abundant and rare subcommunities were significantly correlated to the differences in latitude, MAP, conductivity, pH, and N:P (Figure 5) and the abundant subcommunities were also significantly correlated to the differences in SOC, TN, TP, and C:N (Figure 5).

### Assembly processes

**Figure 6:**
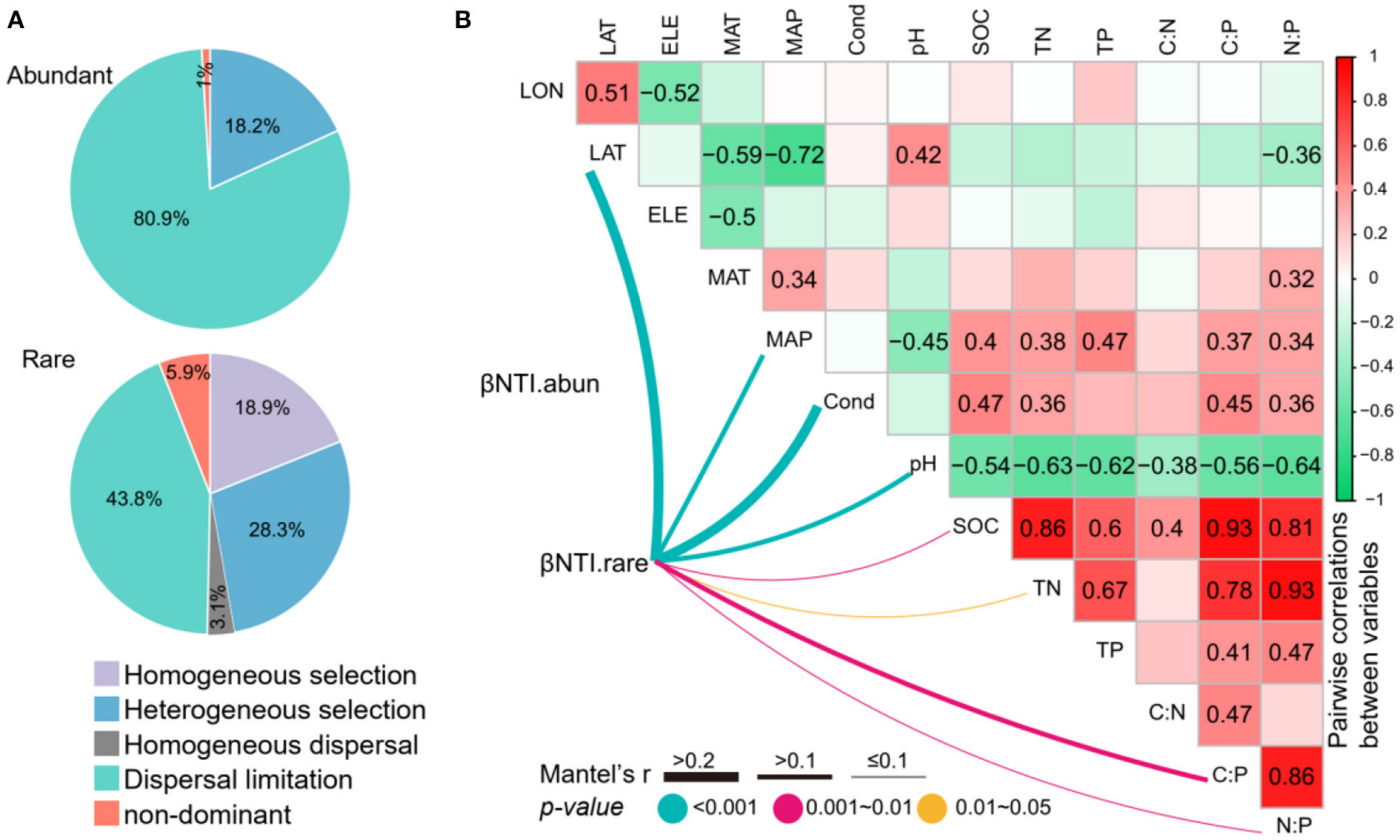
**(A)** The contribution of deterministic (homogeneous and heterogeneous selections) and stochastic (dispersal limitations and homogenizing dispersal) processes to the assembly of abundant and rare subcommunities. **(B)** Pairwise correlations between environmental variables, and the Mantel tests between environmental variables vs. the beta nearest taxon index (βNTI). The lines denote the significant relationships with line width representing the Mantel r statistic. Pairwise correlations between environmental variables are shown in the heatmap matrix denoting Pearson's correlation coefficient. The significant results are shown in numbers. The abbreviations of the environmental variables are explained in the Methods section.

Null model analysis showed that the assembly of abundant subcommunities was dominantly shaped by dispersal limitation processes (80.9%), followed by heterogeneous selection processes (18.2%) and non-dominant processes (1.0%) (Figure 6). However, the rare subcommunity assembly was shaped almost equally by deterministic (18.9% of homogeneous selection and 28.3% of heterogeneous selection) and stochastic (3.1% of homogeneous dispersal, 43.8% of dispersal limitation, and 5.9% of non-dominant) processes (Figure 6). The relationships between βNTI and major environmental variables were used to estimate changes in the relative influences of deterministic and stochastic assembly processes. The Mantel tests showed that the βNTI of abundant subcommunities was not significantly associated with any of the environmental variables. However, the βNTI of rare subcommunities was significantly associated with LAT, MAP, conductivity, pH, SOC, TN, C:P, and N:P (Figure 6), suggesting that the increasing divergences of these variables could contribute to a shift from homogeneous selection to stochastic assembly and heterogeneous selection for the assembly of rare subcommunities.

## Discussion

### General patterns of bacterial diversity and community structure

Our results revealed that the OTU richness of both the abundant and rare subcommunities in thermokarst lake sediment was commonly influenced by location, climate, and nutrient balance, presenting close relationships with latitude, MAP, C:P, and N:P. In general, OTU richness responded more strongly to location and climate variables for abundant subcommunities, while more strongly to physicochemical variables for rare subcommunities. Moreover, both the abundant and rare subcommunities structures had clear distribution patterns along the gradient of latitude and MAP. In thermokarst lakes, the initial sediments are originated from the thaw and collapse of permafrost and then supplemented continuously through horizontal and vertical permafrost degradation (West and Plug, 2008; de Jong et al., 2018). Thus, the bacterial communities in sediments of thermokarst lakes are expected to inherit certain properties of bacterial communities in permafrost soil and also reflect terrestrial–aquatic connections. In ecology, decreasing biodiversity along latitudinal gradients is one of the most fundamental patterns (Willig et al., 2003). In a manner consistent with latitudinal patterns of flora, fauna, and microeukaryotes (Hillebrand, 2004; Peat et al., 2007; Tedersoo et al., 2014), bacterial diversity in soil has been observed to decline with increased latitude in many previous research (Yergeau et al., 2007; Zhang et al., 2018b; Lee et al., 2019). In our study, the OTU richness of both the abundant and rare bacterial subcommunities was negatively correlated to latitude. In addition to latitude, OTU richness of both the abundant and rare subcommunities had positive relationships with MAP, which has been found as another dominant environmental factor regulating bacterial communities and richness, particular in semiarid regions (Bachar et al., 2010; Yuan et al., 2014; Li et al., 2022). A humid climate usually results in higher biodiversity and productivity than a dry climate. In the thermokarst landscape, moreover, surface runoff caused by precipitation could enforce the delivery of terrestrial source bacteria to lake sediment, enriching bacterial diversity and shifting community structure in the sediment. In addition, our study also showed that high bacterial diversity is accompanied by high C:P and N:P. The availability and balance of nutrients have also been demonstrated as essential factors structuring bacterial communities (Torsvik et al., 2002; Lee et al., 2017; Zhou et al., 2020). High organic matter contents can sustain complex microbial communities with high diversity (Garrido-Benavent et al., 2020; Ren and Gao, 2022).

The relative abundance of abundant and rare taxa showed opposite patterns that abundant taxa had greater relative abundance at higher latitude and pH, but at lower MAP, SOC, TN, C:P, and N:P. However, the relative abundance of rare taxa had completely opposite patterns compared to abundant taxa. The results suggested that abundant taxa have high adaptation to unfavorable environments. It has been widely demonstrated that pH is a determinant factor in controlling bacterial diversity and community composition in soil from local to global scales (Fierer and Jackson, 2006; Lauber et al., 2009; Griffiths et al., 2011). Lower precipitation could possibly enrich some stress-tolerant taxa (Li et al., 2022). Moreover, abundant microbial taxa have higher nutrient utilization potential and can easily access limited nutrients (Jia et al., 2018; Zhalnina et al., 2018). Abundant microbial taxa usually exhibit broader response thresholds to environmental factors (Zhalnina et al., 2018; Jiao and Lu, 2020; Wan et al., 2021), suggesting broader environmental adaptations. On the contrary, rare microbial taxa usually have lower competition potential and growth rate, and narrower niche breadth and, thus, exhibit high sensitivity to environmental changes than abundant ones, leading to constrained environmental adaptations (Reveillaud et al., 2014; Pascoal et al., 2021). Thus, rare microbial subcommunities are more controlled by environmental variables, suppressing their abundance under unfavorable environments, such as high pH and latitude and low MAP and nutrients in this study.

### Assembly mechanisms

Microbial community assembly is considered to be governed by both stochastic (dispersal limitation and homogenizing dispersal) and deterministic processes (heterogeneous selection and homogeneous selection) (Stegen et al., 2013; Zhou and Ning, 2017). It is an important topic in microbial ecology to estimate the relative contributions of stochastic and deterministic processes in governing microbial communities (Zhou and Ning, 2017; Ning et al., 2019) because community assembly mechanisms couple microbial community structure with their functions (Graham et al., 2016; Graham and Stegen, 2017; Le Moigne et al., 2020; Luan et al., 2020). Abundant and rare subcommunities might be structured in different assembly regimes (Liu et al., 2015; Pablo Nino-Garcia et al., 2016; Ren and Gao, 2022). In both the aquatic and terrestrial ecosystems, it has been suggested that abundant microbial taxa are more limited by dispersion than rare taxa (Liu et al., 2015; Wu et al., 2016; Jiao and Lu, 2020; Wan et al., 2021). In line with these previous studies, our study also showed that both the abundant and rare subcommunities were dominated by stochastic processes, with abundant subcommunities being influenced more by dispersal limitations than rare subcommunities. However, deterministic processes (homogeneous and heterogeneous selections) also contributed considerably to shaping rare subcommunity assembly. For microbial dispersal, passive dispersal is one of the common mechanisms (Ai et al., 2013; Custer et al., 2022), which is mediated by wind and rain or by hitchhiking on other organisms and could be easily influenced by physical barriers, such as mountains (Prosser et al., 2007; Jia et al., 2018; Mestre and Höfer, 2021). The Qinghai-Tibet Plateau is influenced by the East Asian monsoon, Indian monsoon, and continental westerlies (He et al., 2020), which can carry a variety of microorganisms and disperse across the QTP (Xing et al., 2021; Qi et al., 2022). On the QTP, however, many mountains, such as Tanggula Mountains, Kunlun Mountains, Nyenchen Tanglha Mountains, and Bayan Har Mountains, span in an east-west direction, acting as geographical barriers for both the macro- and microorganisms (Wan et al., 2016; Yu et al., 2019; Ren et al., 2022c). For abundant subcommunities, in particular, it has been hypothesized that abundant taxa can be more easily dispersed than the rare taxa due to higher local population sizes, which guarantee wider dispersal potentials (Nemergut et al., 2011). However, the opposite observation in our study may be explained by an alternative hypothesis that abundant taxa are more affected by dispersal limitation due to wide niche breadth, while rare taxa are more affected by environmental filtering due to narrow niche breadth (Pandit et al., 2009). This alternative hypothesis suggests that the habitat occupancy of habitat generalists (taxa with wide niche breadth) may be governed by the possibility to reach multiple locations (dispersal limitation) (Pandit et al., 2009; Wu et al., 2016). Nevertheless, habitat specialists (taxa with narrow niche breadth) may be strongly governed by environmental selection. In our study, the high contribution of deterministic process in shaping rare subcommunity assembly suggests rare taxa were more strongly controlled by environmental variables than abundant taxa.

To get a deep understanding of microbial assembly mechanisms, it is crucial to disentangle environmental variables affecting the balance between stochastic and deterministic processes (Stegen et al., 2016; Tripathi et al., 2018; Logares et al., 2020). It has been found that in different soil environments, the relative contributions of stochastic and deterministic processes to soil bacterial communities are regulated by different environmental variables, such as pH, salinity, moisture, and nutrients (Tripathi et al., 2018; Zhang et al., 2019; Ren and Gao, 2022). In this study, we observed that βNTI of abundant subcommunity did not associate with any measured environmental variables, further supporting the results that abundant taxa are less environmentally constrained and the stochastic processes are dominant in structuring abundant subcommunities in thermokarst lake sediment across the QTP. On the contrary, βNTI of rare subcommunities had significant relationships with location (latitude), climate (MAP), and physicochemical factors (conductivity, pH, SOC, TN, C:P, and N:P). The results suggest that these environmental factors are the crucial factor in adjusting the balance between stochastic and deterministic processes for rare subcommunities, further validating that rare subcommunities are more environmentally controlled. The divergences of assembly processes between abundant and rare subcommunities might be owing to the differences in their adaptive capabilities to environmental changes (Morrissey et al., 2019; Nyirabuhoro et al., 2021). High environmental heterogeneity across space might impose strong environmental filtering on rare taxa (Langenheder and Lindstrom, 2019; Li et al., 2022). Thus, our results suggest that geographical barriers, climate, and some local physicochemical variables are key factors in shaping the assembly of rare subcommunities.

### Implications for future climate change

With global warming, climate change on the QTP would generally cause increasing temperature and precipitation (Xu et al., 2008; Lu et al., 2018). As a result of climate change, permafrost thawing will be accelerated, converting tremendous permafrost soil to sediments in thermokarst lakes (West and Plug, 2008; de Jong et al., 2018) and releasing terrestrial nutrients and microorganisms into the lake (Bowden, 2010; Biskaborn et al., 2019; Manasypov et al., 2021). Thus, thermokarst lakes on the QTP will undergo substantial changes in their physical, chemical, and biological properties. According to our results, the consequences of future climate change on bacterial communities in thermokarst lake sediment can be presumed. The richness of both the abundant and rare taxa and the relative abundance of rare subcommunities will increase. A stronger contribution of deterministic processes is expected in structuring rare subcommunities.

## Data availability statement

The data presented in the study are deposited in the China National Center for Bioinformation repository, accession number PRJCA009850, CRA007082.

## Author contributions

ZR designed the study, did the analyses, prepared the manuscript, and performed the fieldwork and laboratory work. WL did the visualization and prepared the manuscript. CZ revised the manuscript and did the laboratory analyses. All authors contributed to the article and approved the submitted version.

## Funding

This study was supported by startup funding from the Beijing Normal University and the Key Laboratory of Polar Science, MNR, Polar Research Institute of China.

## Conflict of interest

The authors declare that the research was conducted in the absence of any commercial or financial relationships that could be construed as a potential conflict of interest.

## Publisher's note

All claims expressed in this article are solely those of the authors and do not necessarily represent those of their affiliated organizations, or those of the publisher, the editors and the reviewers. Any product that may be evaluated in this article, or claim that may be made by its manufacturer, is not guaranteed or endorsed by the publisher.
