# Supplementary material for: Rare bacterial biosphere is more environmental controlled and deterministically governed than abundant one in sediment of thermokarst lakes across the Qinghai-Tibet Plateau
Source: Front Microbiol. 2022 Jul 25;13:944646. doi: 10.3389/fmicb.2022.944646 (PMC9358708; doi:10.3389/fmicb.2022.944646)
Supplement: Supplementary file 1 [file Table_1.docx]

## Supplementary Information

Table S1 The basic information of the studied lakes.

| ID | Longitude | Latitude | Elevation  (m) | MAT  (℃) | MAP  (mm) | Conductivity  (us/cm) | pH | SOC  (g/kg) | TN  (g/kg) | TP  (g/kg) | C:N | C:P | N:P |
| --- | --- | --- | --- | --- | --- | --- | --- | --- | --- | --- | --- | --- | --- |
| TS01 | 98.59 E | 34.99 N | 4271 | -2.99 | 378 | 100 | 8.38 | 2.939 | 0.414 | 0.523 | 8.280 | 14.528 | 1.755 |
| TS02 | 98.41 E | 34.83 N | 4185 | -2.52 | 335 | 276 | 7.97 | 11.606 | 1.198 | 0.562 | 11.302 | 53.331 | 4.719 |
| TS03 | 98.28 E | 34.86 N | 4168 | -3.34 | 345 | 147 | 8.13 | 16.416 | 1.720 | 0.370 | 11.135 | 114.648 | 10.296 |
| TS04 | 98.15 E | 34.88 N | 4175 | -2.80 | 328 | 634 | 7.96 | 17.142 | 1.720 | 0.460 | 11.628 | 96.240 | 8.276 |
| TS05 | 98.11 E | 34.74 N | 4186 | -2.34 | 327 | 189 | 8.31 | 4.800 | 0.444 | 0.201 | 12.621 | 61.569 | 4.878 |
| TS06 | 98.06 E | 34.68 N | 4176 | -2.47 | 335 | 338 | 8.34 | 18.787 | 2.053 | 0.327 | 10.677 | 148.493 | 13.908 |
| TS07 | 97.79 E | 34.19 N | 4549 | -4.51 | 491 | 247 | 7.92 | 54.994 | 4.800 | 0.546 | 13.366 | 260.213 | 19.469 |
| TS08 | 97.57 E | 34.04 N | 4588 | -5.54 | 571 | 311 | 7.69 | 71.188 | 6.007 | 0.625 | 13.825 | 294.092 | 21.272 |
| TS09 | 97.29 E | 33.92 N | 4433 | -3.66 | 536 | 271 | 7.68 | 81.474 | 7.307 | 0.602 | 13.008 | 349.525 | 26.871 |
| TS10 | 97.47 E | 33.29 N | 4393 | -0.41 | 530 | 605 | 7.55 | 182.708 | 14.047 | 0.680 | 15.175 | 694.594 | 45.773 |
| TS11 | 97.37 E | 33.35 N | 4358 | -0.64 | 529 | 304 | 7.80 | 44.499 | 3.846 | 0.403 | 13.500 | 285.409 | 21.142 |
| TS12 | 97.12 E | 33.8 N | 4175 | -2.26 | 500 | 85 | 8.20 | 22.439 | 2.458 | 0.563 | 10.651 | 103.011 | 9.671 |
| TS13 | 97.16 E | 33.76 N | 4223 | -2.38 | 503 | 128 | 7.81 | 27.254 | 3.325 | 0.674 | 9.562 | 104.464 | 10.925 |
| TS14 | 96.65 E | 32.9 N | 4287 | -1.96 | 669 | 368 | 8.20 | 174.052 | 12.918 | 0.702 | 15.719 | 640.556 | 40.751 |
| TS15 | 96.39 E | 32.48 N | 3831 | 0.94 | 638 | 334 | 7.88 | 28.611 | 3.399 | 0.532 | 9.820 | 138.985 | 14.153 |
| TS16 | 96.52 E | 32.16 N | 3569 | -0.61 | 736 | 101 | 8.22 | 5.037 | 0.739 | 0.424 | 7.952 | 30.705 | 3.861 |
| TS17 | 97.08 E | 30.59 N | 4279 | -1.33 | 717 | 135 | 8.13 | 22.583 | 2.449 | 0.489 | 10.758 | 119.185 | 11.078 |
| TS18 | 96.72 E | 29.65 N | 4394 | 0.18 | 580 | 59 | 8.26 | 3.850 | 0.542 | 0.187 | 8.284 | 53.054 | 6.405 |
| TS19 | 92.37 E | 29.81 N | 4893 | -1.69 | 686 | 29 | 8.12 | 9.544 | 1.033 | 0.498 | 10.778 | 49.549 | 4.597 |
| TS20 | 92.35 E | 29.79 N | 4773 | -2.91 | 701 | 74 | 7.46 | 33.962 | 2.046 | 0.525 | 19.367 | 167.226 | 8.635 |
| TS21 | 91.85 E | 29.77 N | 3871 | 0.81 | 586 | 98 | 7.43 | 48.588 | 3.522 | 0.625 | 16.093 | 200.957 | 12.487 |
| TS22 | 90.59 E | 30.17 N | 4442 | -1.09 | 526 | 564 | 7.11 | 137.490 | 11.705 | 0.604 | 13.704 | 588.046 | 42.911 |
| TS23 | 90.79 E | 30.3 N | 4303 | 1.11 | 494 | 35 | 7.46 | 17.615 | 13.668 | 0.678 | 1.504 | 67.112 | 44.637 |
| TS24 | 91.13 E | 30.49 N | 4232 | 0.84 | 503 | 869 | 8.83 | 34.491 | 2.887 | 0.492 | 13.937 | 181.016 | 12.988 |
| TS25 | 91.61 E | 30.79 N | 4610 | -3.11 | 616 | 567 | 7.70 | 58.714 | 5.153 | 0.565 | 13.294 | 268.392 | 20.189 |
| TS26 | 91.7 E | 31.1 N | 4716 | -1.89 | 512 | 216 | 7.92 | 62.627 | 5.240 | 0.829 | 13.944 | 195.200 | 13.999 |
| TS27 | 91.76 E | 31.17 N | 4585 | -1.89 | 510 | 257 | 7.91 | 41.560 | 3.475 | 0.247 | 13.952 | 435.527 | 31.217 |
| TS28 | 91.85 E | 31.31 N | 4510 | -0.45 | 448 | 416 | 7.92 | 33.443 | 3.501 | 0.319 | 11.145 | 270.467 | 24.267 |
| TS29 | 91.96 E | 31.41 N | 4442 | -0.76 | 447 | 148 | 7.88 | 31.109 | 2.293 | 0.239 | 15.830 | 336.703 | 21.269 |
| TS30 | 91.95 E | 31.55 N | 4540 | -1.44 | 460 | 96 | 8.18 | 21.546 | 2.038 | 0.324 | 12.337 | 172.013 | 13.943 |
| TS31 | 91.8 E | 31.58 N | 4497 | -1.50 | 465 | 576 | 7.74 | 60.521 | 6.311 | 0.558 | 11.188 | 280.095 | 25.036 |
| TS32 | 91.82 E | 31.72 N | 4625 | -2.31 | 480 | 101 | 8.15 | 18.485 | 1.367 | 0.319 | 15.780 | 149.506 | 9.474 |
| TS33 | 91.7 E | 32 N | 4650 | -1.74 | 463 | 145 | 7.96 | 27.456 | 2.555 | 0.244 | 12.536 | 290.775 | 23.195 |
| TS34 | 91.75 E | 32.23 N | 4588 | -1.49 | 450 | 102 | 8.60 | 5.565 | 0.671 | 0.212 | 9.680 | 67.853 | 7.009 |
| TS35 | 91.86 E | 32.66 N | 4959 | -4.01 | 467 | 58 | 8.78 | 0.920 | 0.235 | 0.149 | 4.566 | 15.908 | 3.484 |
| TS36 | 91.91 E | 33.09 N | 4913 | -5.49 | 476 | 140 | 8.27 | 10.617 | 1.173 | 0.283 | 10.561 | 97.004 | 9.186 |
| TS37 | 92.4 E | 34.17 N | 4603 | -2.50 | 291 | 92 | 8.49 | 7.992 | 0.944 | 0.243 | 9.875 | 84.895 | 8.597 |
| TS38 | 92.22 E | 33.77 N | 4570 | -2.86 | 302 | 200 | 8.05 | 12.104 | 1.120 | 0.394 | 12.609 | 79.438 | 6.300 |
| TS39 | 92.73 E | 34.46 N | 4526 | -2.73 | 306 | 212 | 8.50 | 1.904 | 0.294 | 0.154 | 7.562 | 31.998 | 4.232 |
| TS40 | 92.93 E | 34.82 N | 4584 | -3.03 | 296 | 111 | 8.22 | 8.107 | 0.423 | 0.350 | 22.381 | 59.798 | 2.672 |
| TS41 | 92.96 E | 34.96 N | 4525 | -3.30 | 291 | 62 | 9.02 | 1.451 | 0.345 | 0.188 | 4.900 | 19.968 | 4.075 |
| TS42 | 93.33 E | 35.33 N | 4488 | -2.92 | 279 | 149 | 8.81 | 4.547 | 0.649 | 0.337 | 8.178 | 34.902 | 4.268 |
| TS43 | 93.68 E | 35.49 N | 4469 | -2.64 | 304 | 595 | 8.42 | 3.626 | 0.328 | 0.352 | 12.909 | 26.646 | 2.064 |
| TS44 | 93.87 E | 35.52 N | 4572 | -4.00 | 320 | 364 | 8.30 | 4.702 | 0.528 | 0.278 | 10.389 | 43.677 | 4.204 |
